# The Noncollinear Path to Two-Dimensional Topological Superconductivity

**DOI:** 10.1021/acsnano.5c07565

**Published:** 2025-10-09

**Authors:** Reiner Brüning, Jasmin Bedow, Roberto Lo Conte, Kirsten von Bergmann, Dirk K. Morr, Roland Wiesendanger

**Affiliations:** † Department of Physics, 14915 University of Hamburg, 20355 Hamburg, Germany; ‡ Department of Physics, 14681 University of Illinois Chicago, Chicago, Illinois 60607, United States; § Zernike Institute for Advanced Materials, 332143 University of Groningen, 9747 AG Groningen, The Netherlands

**Keywords:** spin spirals, noncollinear magnet-superconductor hybrids, topological nodal-point superconductors, chiral edge modes, spin-polarized scanning tunneling spectroscopy

## Abstract

Two-dimensional magnet-superconductor hybrids (2D-MSH) are promising candidates to realize devices for topology-based quantum technologies and superconducting spintronics. So far, studies have focused on 2D-MSH systems with collinear ferro- or antiferromagnetic layers. Here, we present the discovery of topological superconductivity in a noncollinear MSH system where a magnetic spiral is realized in an Fe monolayer proximity coupled to a superconducting Ta(110) substrate. By combining low-temperature spin-polarized scanning tunneling spectroscopy with an in-depth theoretical study, we can conclude that the system is in a topological nodal-point superconducting phase with low-energy edge modes. Furthermore, we reveal that for this noncollinear spin texture, these edge modes exhibit a magnetization direction-dependent dispersion. This means that a spatial shift of the magnetic spiral could be used to reverse the chirality of an edge mode in future MSH-based devices.

## Graphical abstract

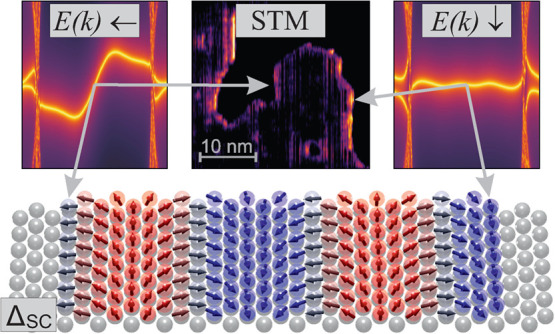

## Introduction

Topological superconductivity in bulk as well as in low-dimensional hybrid structures has become a very active field of research.^1–3^ Early attempts to establish topological superconductivity in 2D-MSH focused primarily on ferromagnetic films in proximity to *s*-wave superconductors.^4–6^ Such systems, in combination with spin–orbit coupling (SOC), are expected to generate a topological phase with a hard superconducting gap and dispersing chiral edge modes. This topological phase can be classified by a topological invariant, the Chern number, which relates to the number of chiral edge modes.^7^ More recently, the attempts to establish topologically nontrivial states have been extended to collinear antiferromagnetic states in two-dimensional structures on conventional superconductors.^8–12^ In these systems, new types of topological phases can emerge such as topological nodal point superconductivity^9,11,12^ and topological crystalline superconductivity.^10^ All of the above-mentioned studies have established that 2D-MSH systems provide a powerful approach for realizing novel topological phases of matter.

While so far, only 2D-MSH systems with collinear ferromagnetic and antiferromagnetic spin textures—a small subgroup of all the possible spin textures available—have been investigated as a source of nontrivial topology, there have been several theoretical predictions of novel topological superconducting phases in noncollinear^13–15^ and noncoplanar^16–18^ spin textures in proximity to a superconductor. However, experimental confirmation of these predictions has been lacking so far.

Here, we report the experimental discovery of topological nodal-point superconductivity in a noncollinear 2D-MSH system, where a single atomic layer of iron (Fe) is grown on top of a clean superconducting Ta(110) substrate. The presence of Ta, with large SOC, is crucial for the stabilization of a cycloidal spin spiral ground state in the Fe monolayer due to the Dzyaloshinskii–Moriya interaction.^19^ Using scanning tunneling microscopy (STM) and spectroscopy, we identify the existence of the spin spiral and gain insight into the spatially resolved electronic structure of the hybrid system by measuring the local differential tunneling conductance, d*I*/d*V*, using both spin-polarized and nonmagnetic tips. Complementary theoretical studies allow us to identify the existence of a topological nodal point superconducting (TNPSC) phase in the system and its characteristic experimental signatures. In particular, we reveal the existence of a topologically protected edge mode along the [001]-edge of a magnetic island, which is absent along the [11̅0]- and [11̅1]-edges. A comparison of these results with the experimentally measured d*I*/d*V* signal along island edges yields good agreement, providing strong evidence for the existence of a TNPSC phase arising from a noncollinear spin texture. Furthermore, we identify direct signatures of Rashba spin–orbit coupling in the experimentally measured differential tunneling conductance and prove its importance for the formation of the topological nodal-point superconducting phase. Moreover, we demonstrate that the dispersion of the edge mode and its spectral weight depend sensitively on the magnetization direction at the island edges. A termination-dependent edge mode is a unique feature of the spiral magnetic structure, not seen before in any of the MSH systems possessing collinear magnetic ground states.

## Results and Discussion

### Spin Spirals in Fe Monolayers on Ta(110)

An overview spin-polarized (SP-)­STM image with about 80% coverage of Fe on Ta(110) is shown in Figure 1a. The Fe monolayer (orange) grows pseudomorphically, and the stripes along the [001] direction are of magnetic origin, visible due to the spin-polarized tip used in this measurement.^20,21^ We identify a spin spiral as the magnetic ground state of the Fe monolayer propagating along [11̅0] with a period of about 6 nm (see ref 22 for more information on the magnetic state). A closer view of the spin spiral is shown in Figure 1b, where bright areas are parallel to the tip magnetization direction, and dark areas are antiparallel to it (or vice versa). This imaging mechanism is based on the tunnel magnetoresistance (TMR) effect and the signal scales with the cosine of the angle enclosed by tip and sample magnetization, i.e., it directly reflects the magnetic periodicity. This is shown in Figure 1c,d, where the expected imaging contrast is directly compared to a sketch of the respective spin spiral.

**Figure 1:**
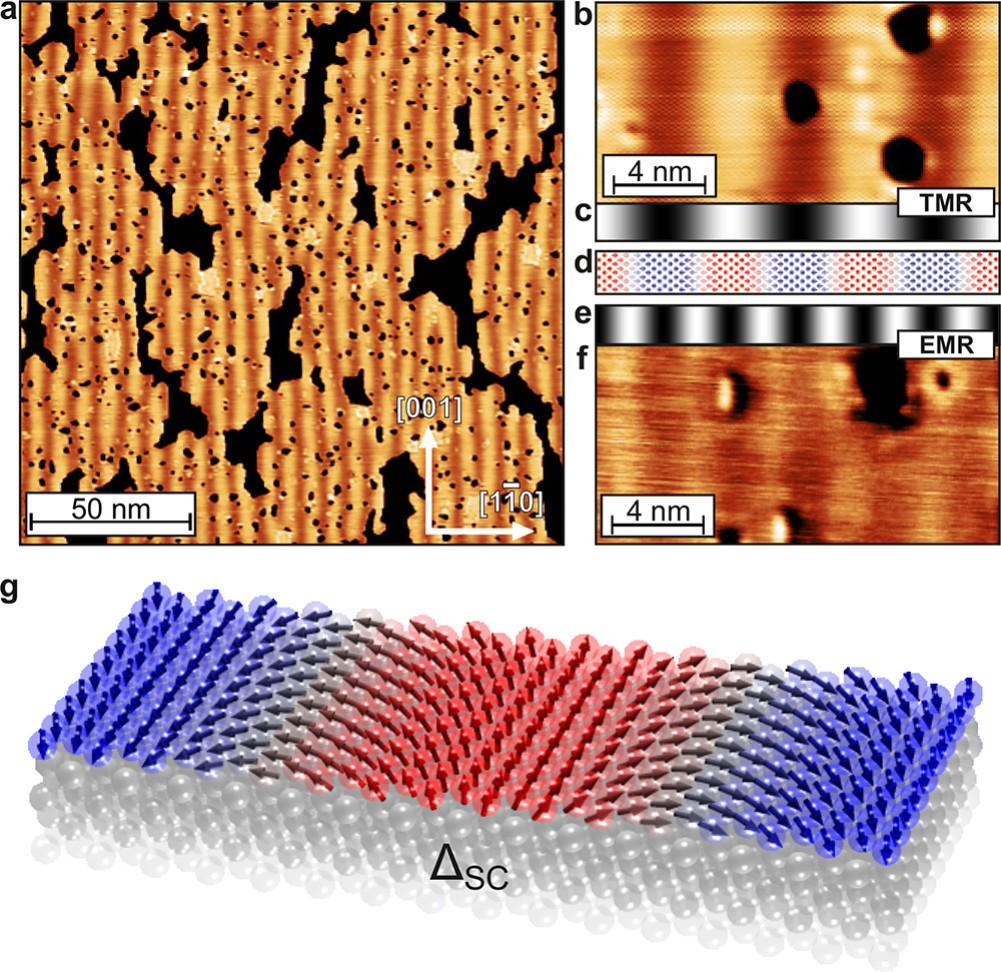
Magnetic characterization of the MSH system. (a) SP-STM constant-current image of a sample with 0.8 atomic layers of Fe on Ta(110); the pseudomorphic Fe monolayer areas can be identified by their orange color, and the stripes originate from the magnetic spin spiral state. (b) Closer view of an SP-STM constant-current measurement of the spin spiral in the Fe monolayer. (c) Expected SP-STM signal due to the TMR effect for a spin spiral. (d) Sketch of a homogeneous spin spiral; red and blue indicate up and down magnetization directions, respectively. (e) Expected signal for a spin spiral using a nonmagnetic tip due to EMR effects. (f) STM constant-current image obtained using a nonmagnetic tip, revealing a periodic pattern with half of the spin spiral period, in agreement with EMR contrast. (g) Perspective view of the studied MSH system.

When a nonmagnetic tip is used, as for the STM image displayed in Figure 1f, a stripe pattern with half the spin spiral period becomes visible, albeit with much lower intensity. This has previously been observed in spin spiral systems and has been ascribed to either a SOC-induced tunnel anisotropic magnetoresistance (TAMR)^23,24^ or—in the case of inhomogeneous spin spirals—to a noncollinear magnetoresistance (NCMR) originating from spin mixing^25–27^ (see expected imaging contrast in Figure 1e, Supplementary Note 1, and Supplementary Figure S1). Because we cannot distinguish experimentally which of the two effects is dominating in our system, we refer to this observation generally as an electronic magnetoresistance (EMR) effect in the following, as both effects are related to slight changes of the local electronic states due to the noncollinearity of the spin texture. A perspective view of the established 2D-MSH model system is displayed in Figure 1g.

### Low-Energy State Oscillations of the Superconducting Fe/Ta(110) Hybrid System

A sketch of the magnetic spin texture with the expected signal due to the TMR effect is displayed again in Figure 2a, while in Figure 2b, we present the spin-resolved d*I*/d*V* measured as a function of the sample bias and the position within the spin spiral. These measurements reveal the existence of a superconducting gap in the Fe monolayer, with the coherence peaks indicated by the purple dashed lines at ±Δ_s_. Moreover, we observe a modulation of the d*I*/d*V* intensity with the spin spiral period not only outside the superconducting gap, i.e., due to the TMR as in Figure 1a,b, but also for the coherence peaks. The intensity oscillations of the positive and negative energy coherence peaks are different, indicating that the coherence peaks have different spin polarizations.

**Figure 2:**
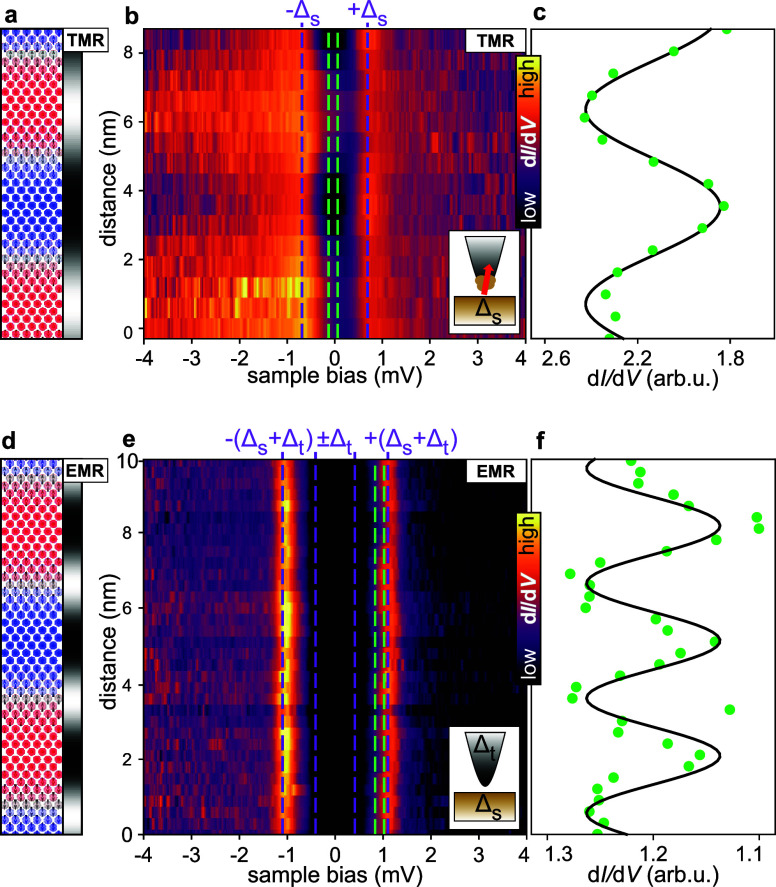
Spectroscopic characterization of the MSH system. (a) Spin configuration of the magnetic spin spiral and the expected imaging contrast due to the TMR effect. (b) Spin-resolved d*I*/d*V* spectroscopy measurements were obtained with a spin-polarized tip along the spin spiral propagation direction. (c) d*I*/d*V* intensities along the spin spiral averaged over an energy range of −0.13 to +0.06 mV, as indicated by the green lines in (b); the solid line represents a cosine function with the spin spiral period and serves as a guide to the eye. (d) Spin configuration of the magnetic spin spiral and the expected imaging contrast due to the EMR effect. (e) Spin-averaged d*I*/d*V* spectroscopy measurements were obtained with a superconducting tip along the spin spiral. (f) d*I*/d*V* intensities along the spin spiral averaged over an energy range of +0.80 to +1.02 mV, as indicated by the green lines in (e); the cosine function with half the spin spiral period serves as a guide to the eye.

In Figure 2c, we present a line-cut of the d*I*/d*V* signal averaged over a narrow bias voltage window around zero bias (see the green dashed lines in Figure 2b); it reveals the signature of the spin spiral periodicity even deep inside the superconducting gap, exhibiting an approximate cosine dependence (see the solid line in Figure 2c). More details are available in Figure S2. We interpret this as a manifestation of the spin-polarization of the low-energy states.

To further investigate the nature of the in-gap electronic states in the studied MSH system, we employ a nonmagnetic superconducting tip consisting of a Cr bulk tip with a superconducting Ta-cluster at its apex. Figure 2e displays the measured d*I*/d*V* signal as a function of sample bias and position within the spin spiral (see Figure 2d for the expected EMR signal with a nonmagnetic tip). Due to the superconducting tip, the coherence peaks are now shifted to an energy ±(Δ_s_ + Δ_t_) (outer purple dashed lines; the inner purple dashed lines indicate the tip gap ± Δ_t_). To better visualize the weak spatial variations of the d*I*/d*V* intensity in Figure 2e, we average the signal over a small bias range near the positive bias coherence peak (see green dashed lines). The resulting d*I*/d*V* signal shown in Figure 2f reveals a periodicity, which is half of that of the spin spiral (for additional data, refer to Figure S3). Although the signal variation is much weaker compared to the spin-polarized case, this result demonstrates that the spin spiral induces an EMR-like contrast with half the magnetic wavelength, even for states inside the superconducting gap.

### Theoretical Analysis of the Origin of the Low-Energy State Oscillations

To obtain a better understanding of the physics emerging from the coexistence of spin spiral order and superconductivity, and the microscopic electronic origin of the TMR and EMR signals, we model the MSH system using a minimal Hamiltonian of the form

$$H=-\mu \sum_{r,\alpha }c_{r,\alpha }^{†}c_{r,\alpha }+\Delta \sum_{r}(c_{r,↑}^{†}c_{r,↓}^{†}+c_{r,↓}c_{r,↑})+\sum_{r,r^{\prime },\alpha }t_{r,r\prime }c_{r,\alpha }^{†}c_{r^{\prime },\alpha }+J\sum_{r,\alpha ,\beta }c_{r,\alpha }^{†}[S(r)\cdot \sigma {]}_{\alpha ,\beta }c_{r,\beta }-i\sum_{r,r^{\prime },\alpha ,\beta }\alpha_{r,r^{\prime }}c_{r,\alpha }^{†}{\left({ê}_{r-r^{\prime }}\times \sigma \right)}_{\alpha ,\beta }^{z}c_{r^{\prime },\beta }$$ 1

where *c*_**r**, α_^†^ creates an electron of spin α at site **r**, μ is the chemical potential, and Δ is the *s*-wave superconducting order parameter. We denote by *t*_**r**,**r’**_ the electronic hopping amplitudes with different values for nearest (*t*_0_), next-nearest (*t*_1_), and next–next-nearest neighbor (*t*_2_) sites. *J* is the magnetic exchange coupling between the spins of the Fe film and that of the conduction electrons. α_**r**,**r’**_ is the Rashba spin–orbit (RSO) coupling strength between electrons at sites **r** and **r**′, where we consider the same sets of bonds as for the hopping, which arises from the broken inversion symmetry on the surface.

To identify the microscopic origin of the experimental TMR and EMR signals, we consider two different cases: a homogeneous (sinusoidal) spin spiral (a) without RSO (this case had previously been considered in ref 13) and (b) with a nonzero RSO. In Figure 3a,b, we present the corresponding out-of-plane magnetization, *m*_*z*_, of the spin spiral (blue), together with the resulting zero-energy spin-resolved (orange) and spin-averaged (green) LDOS for these two cases along a line-cut parallel to the spiral propagation direction **Q**. We note that the spin-resolved and spin-averaged LDOS are directly related to the experimentally obtained d*I*/d*V* signals with a spin-polarized (TMR) and a nonmagnetic (EMR) tip, respectively. Moreover, the noncollinear nature of the spin structure induces an additional Rashba spin–orbit interaction, RSO_i_ , such that topological superconductivity can arise even in the absence of a conventional RSO.^13^ ,^16^ ,^17^ The induced RSO_i_ in combination with the conventional RSO leads to a total RSO_t_ , shown as a red line in Figure 3a,b, that determines the emerging properties of the system.

**Figure 3:**
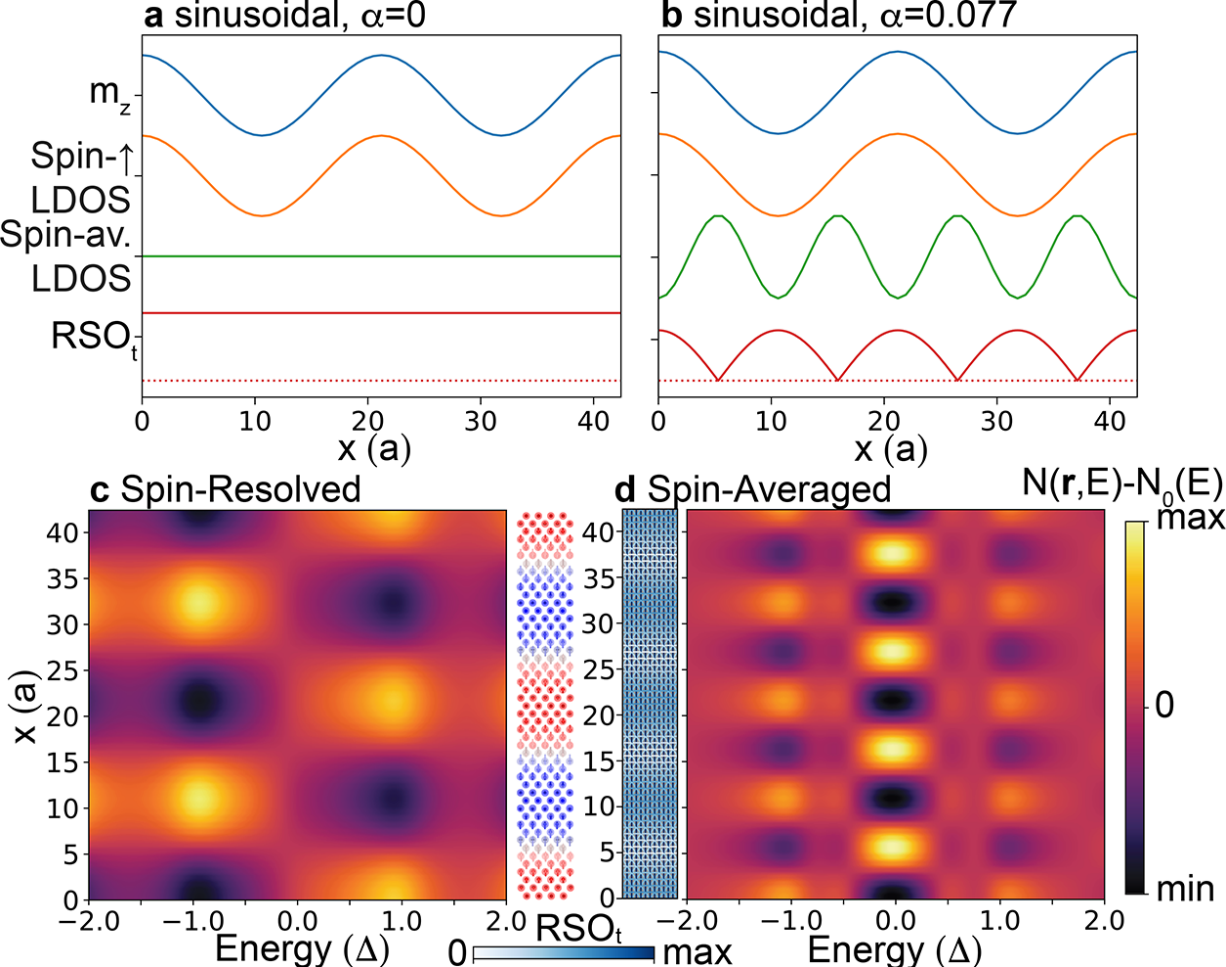
Microscopic origin of the TMR and EMR signals. Line-cut of the out-of-plane magnetization (blue), spin-*↑* LDOS (orange), spin-averaged LDOS (green), and total Rashba SOC (red) for a sinusoidal spin spiral with (a) α = 0 and (b) α = 0.077Δ; RSO_*t*_ = 0 is indicated by the red dotted lines. For the case shown in (b), the energy- and position-dependent (c) spin-resolved and (d) spin-averaged LDOS (with the spatially averaged LDOS for each energy *N*_0_(*E*) subtracted) for a line-cut along the spiral direction of propagation, together with the spatial dependence of *m*_*z*_ and the total Rashba SOC.

For both cases, the spin-resolved LDOS follows the spatial form of *m*_*z*_, thus capturing the experimentally observed spin spiral period, as observed via TMR (see Figure 2c). In contrast, the spin-averaged LDOS reflects the spatial form of RSO_t_. This leads to a spatially constant LDOS for case (a), in disagreement with the experimentally observed EMR signal (Figure 2f). However, for case (b), the spin-averaged LDOS exhibits a spatial modulation with a periodicity of half of the spin spiral, in agreement with the experimental EMR results. We note that for the case of Figure 3b, the complex interplay between the conventional and the induced RSO leads to this spatially modulated RSO_t_ (for further details, see the Methods section). We thus attribute the origin of the experimentally observed EMR signal to the interplay between the RSO coupling and the spin spiral. While we explore the consequences of this interplay further below, we note that an alternative origin of the EMR signal could be given by the presence of an inhomogeneous spin spiral even in the absence of an RSO coupling (see Figures S4 and S5). Both models, a homogeneous spin spiral with conventional RSO or an inhomogeneous spin spiral without RSO coupling, lead to qualitatively the same results for our system. In the following, we focus on the first scenario.

In Figure 3c,d, we present a line-cut of the energy-dependent spin-resolved and spin-averaged LDOS, respectively, along **Q** (to highlight the spatial oscillation for both cases, we have subtracted the respective spatially averaged LDOS, N_0_(E)). In the spin-resolved case, we find that the LDOS at the position of the coherence peaks is modulated with the spin spiral period, with an enhancement of the LDOS at one coherence peak accompanied by suppression at the other. This pattern agrees well with the oscillatory pattern of the TMR signal at ±Δ_*s*_ shown in Figure 2b. The spin-averaged LDOS, Figure 3d, also shows a spatially oscillating pattern, however, with a period that is half of that of the spin spiral, as observed experimentally with a nonmagnetic tip (Figure 2f). While the EMR signal oscillations in the experimental data are observed only in a small bias voltage regime, in our calculations, they are present over the entire gap region. In addition, the theoretical results exhibit a complementary intensity pattern between the coherence peaks and the low-energy region around *E*_*F*_, with the region of largest RSO_t_ interaction coinciding with the lowest LDOS at *E*_*F*_ (see the left side of the image in Figure 3d). These results suggest that the experimentally accessible spin-averaged LDOS in the superconducting gap reflects the complex spatially dependent total RSO_t_ , a parameter that governs the emerging properties of noncollinear MSH systems.

### Spectroscopic Study of Edge Modes at the Boundaries of Fe Islands

Next, we explore the spectroscopic signatures along the edges of the Fe islands, as shown in Figure 4a, where we present a d*I*/d*V* map obtained at *V* = 0.1 mV for one of the investigated Fe islands. The observed strongly enhanced intensity in particular at the [001]-edge raises the intriguing question of whether it is related to the existence of a topological edge mode, as has previously been found in collinear MSH systems.^9^ To investigate this question, we plot in Figure 4d the calculated electronic band structure of the system, which exhibits several nodal points in the magnetic Brillouin zone (the corresponding magnetic unit cell in real space is shown in Figure 4b). As these nodal points possess a quantized topological charge, *q* = ±1 (for details, see Methods section and Figure S6), we conclude that the system is in a topological nodal point superconducting (TNPSC) phase. As a result, the LDOS of the bulk Fe/Ta system exhibits a characteristic *V*-shape around zero energy, directly reflecting the existence of these nodal points, in contrast to the uncovered Ta surface, where the LDOS exhibits a hard *s*-wave superconducting gap (see Figure 4c).

**Figure 4:**
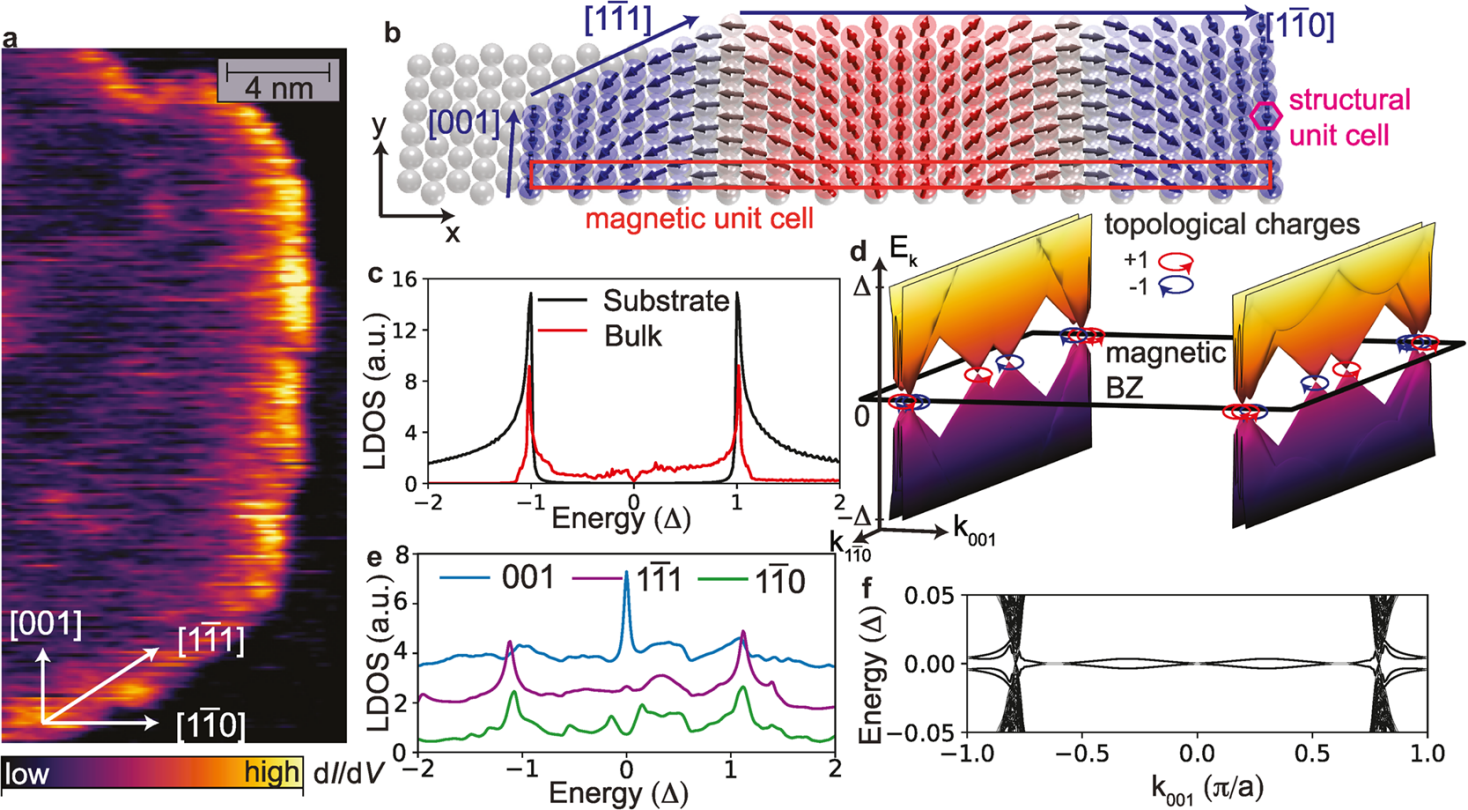
Topological nodal point superconductivity and edge modes in the MSH system. (a) Spin-averaged in-gap d*I*/d*V* map at *V* = 0.1 mV exhibiting an enhanced contrast along the [001]-edge. (b) Sketch of the structural and magnetic unit cell. (c) Theoretical LDOS on the Ta surface (black) and the Fe/Ta MSH system (red). (d) Electronic structure in the magnetic Brillouin zone exhibiting nodal points with nonzero topological charge. (e) Theoretical LDOS at a [001]-edge (blue), [11̅1]-edge (purple), or [11̅0]-edge (green). (f) Electronic band structure as a function of momentum along the [001]-edges of a ribbon system. The spin spiral terminates with an angle θ = 0° at the ribbon’s left and right edges.

A unique feature of the TNPSC phase is that it is associated with zero energy modes only along certain real space edges, which are determined by the interplay of the nodal point position in momentum space, their projection onto the edge direction, and the magnetic structure of the edge.^9,11,12^ Fe/Ta(110) islands, in general, realize three different types of edges along the [001], [11̅1], and [11̅0] directions, as shown in Figure 4b. A comparison of the LDOS for these three edges (see Figure 4e) reveals that a zero-energy peak occurs only along the [001]-edge but is absent for the other two edges (for details, see Figure S6). This peak arises from a low-energy, weakly dispersing chiral edge mode that connects nodal points of opposite topological charge, as can be seen in the plot of the electronic band structure of a ribbon system with [001]-edges, shown in Figure 4f. This finding suggests that the enhanced d*I*/d*V* along the edges of the Fe islands shown in Figure 4a (and Figure S7) reflects the existence of a chiral edge mode.

### Influence of the Noncollinearity of the Spin Texture on the Edge Mode Dispersion

Until now, chiral edge modes have been observed only in collinear MSH systems. In our noncollinear case, the termination of the cycloidal spin spiral by a [001]-edge can lead to an arbitrary angle θ between the spin direction at the edge and the surface normal, as schematically shown for two values of θ in Figure 5a,b. This naturally raises the question of whether the termination angle θ has any effect on the properties of the edge mode. To investigate this question, we present in Figure 5a,b the momentum- and energy-resolved spectral function at a ribbon’s left edge for two different termination angles of θ = 172° and θ = 82°, respectively. These results demonstrate that the edge mode dispersion can indeed be changed by varying θ, with the least and most dispersive edge modes obtained for the cases shown in Figure 5a,b.

**Figure 5:**
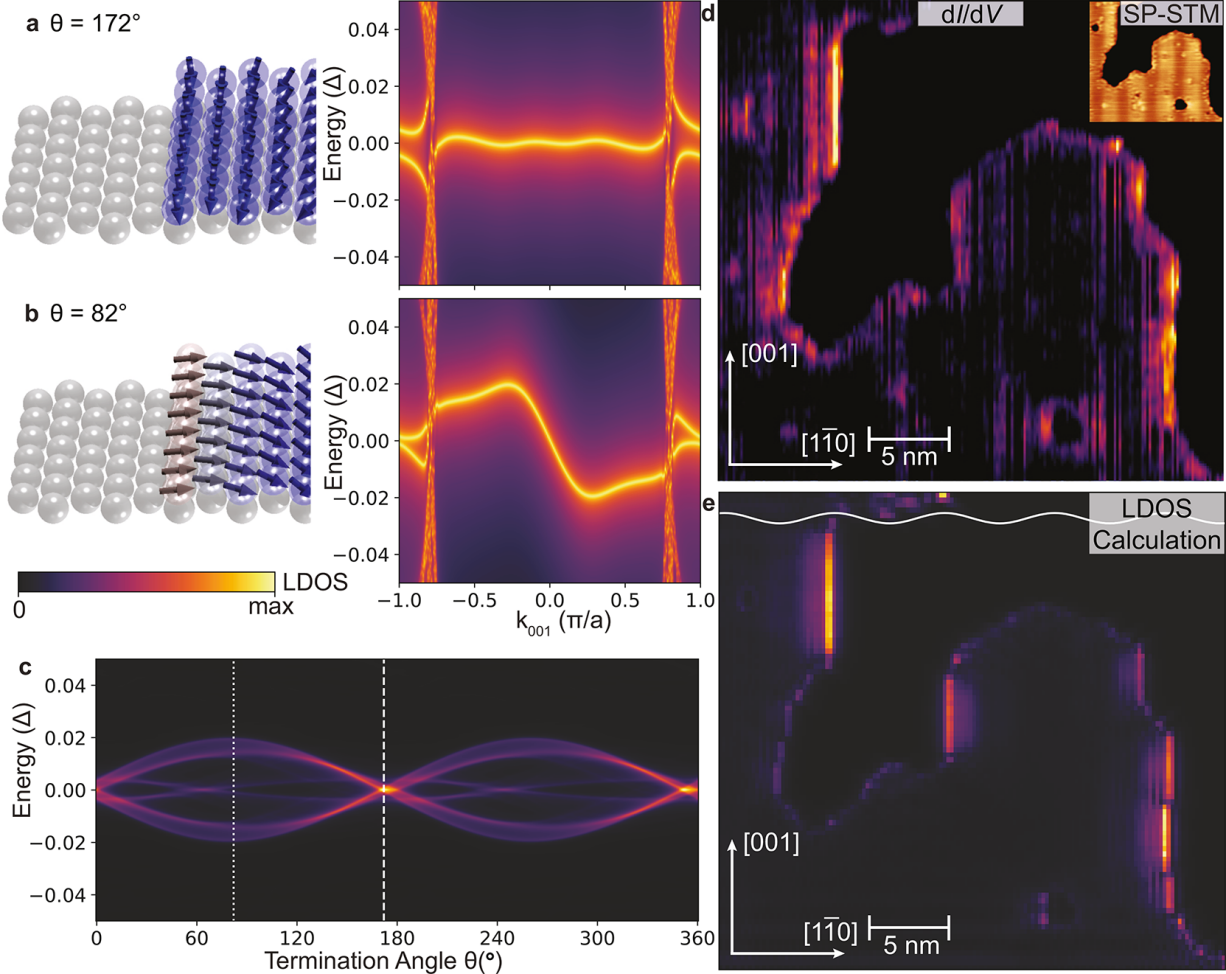
Edge states along the [001] direction. Spectral function as a function of momentum along a ribbon with two [001]-edges and termination angle (a) θ = 172° and (b) θ = 82° of the spin spiral. (c) Low-energy LDOS at an [001]-edge as a function of the termination angle θ. The white dashed and dotted lines represent the θ = 172° and θ = 82° terminations, respectively. (d) Experimental spin-averaged zero-bias d*I*/d*V* map with a fast scan direction along [001]-edges of an irregularly shaped Fe island; the inset shows an SP-STM constant-current image of the same area. (e) Theoretical spin-averaged zero-energy LDOS for a Fe island of the same size and shape as that shown in (d). The theoretical LDOS data were convoluted in energy with a Lorentzian of width Δ*E* = 0.01Δ. The white line represents the *m*_*z*_-component of the spin spiral along the [11̅0]-direction.

This change in the dispersion with varying θ is directly reflected in the form of the low-energy LDOS, as shown in Figure 5c, where we present the energy-resolved LDOS at a [001]-edge as a function of θ. While for θ = 172°, the LDOS exhibits a strong peak at zero-energy, corresponding to the very weakly dispersing edge mode shown in Figure 4f, the more dispersive edge mode for θ = 82° results in two peaks in the edge LDOS at nonzero energy and a much weaker LDOS at zero energy. This termination-dependent edge mode is a unique feature of the spiral magnetic structure not seen before in any of the MSH systems possessing collinear magnetic ground states. This feature could in principle be used to reverse the chirality of an edge mode, by sliding the spiral across the systems such that the termination angle changes by 180° (for details, see Figure S8).

These intriguing results—the existence of a low-energy chiral edge mode at a [001]-edge and the absence thereof at [11̅1]- and [11̅0]-edges, as well as a variation in intensity of the zero-energy LDOS at a [001]-edge with the termination angle—are unique and experimentally observable features of this TNPSC phase. To test these theoretical predictions, we study an Fe island (whose SP-STM image is shown in the inset of Figure 5d) that possesses several [001]- and [11̅1]-edges. A measurement of the spin-averaged zero-bias d*I*/d*V*, shown in the main panel of Figure 5d, reveals two main results: a large intensity along the [001]-edges, which varies between different [001]-edges, and a very weak intensity along the [11̅1]-edges. These findings provide strong evidence for the existence of a TNPSC phase and for the predicted termination angle dependent zero-energy LDOS at [001]-edges shown in Figure 5c. To directly compare our experimental findings with our theoretical model, we computed the spin-averaged zero-energy LDOS for an Fe island of the same size and shape as the experimental one, as shown in Figure 5e. A comparison of the experimental d*I*/d*V* in Figure 5d with the theoretical LDOS in Figure 5e shows very good agreement, both in terms of a large intensity along [001]-edges only and a variation in intensity along different [001]-edges, providing further support for the existence of a TNPSC phase in Fe/Ta(110). The presence of these edge modes in spite of the finite edge disorder exhibited by the Fe island demonstrates the robustness of the nodal point topological phase against disorder effects. We note that in the absence of a TNPSC phase, i.e., for a trivial system, edge disorder cannot give rise to a low-energy edge mode (see Figure S11), as the effective time-reversal symmetry of the system prohibits the creation of in-gap states.

## Conclusion

In conclusion, our combined experimental and theoretical study of the spin spiral state of an Fe monolayer proximity coupled to a superconducting Ta(110) substrate revealed an enhanced LDOS at [001]-edges of Fe islands, which can be attributed to a chiral edge mode resulting from topological nodal-point superconductivity. Due to the noncollinearity of our spin texture, different magnetization directions can occur at the structurally identical [001]-edges, which strongly affects the dispersion of the chiral edge mode. We expect that our finding of magnetization-direction-dependent chiral edge modes can be used as an additional knob for future application concepts in the realm of topological superconductivity and superconducting spintronics.

## Methods

### Experimental Details

To obtain a clean Ta(110) surface, the single crystal was flashed by electron beam heating up to ∼2200 °C multiple times for 60 s. The Fe was evaporated from a rod and deposited on top of the crystal shortly after the flash by physical vapor deposition under ultrahigh-vacuum conditions with a base pressure of ∼1.0 × 10^–10^ mbar.

The samples were transferred in situ to a home-built STM system operated at 1.3 or 4.2 K. All STM measurements presented in this work were performed using a bulk Cr tip. To obtain a superconducting spin-averaging tip, we modified the apex with superconducting Ta clusters from the clean Ta surface (EMR-related measurements).

The d*I*/d*V* measurements were performed using a lock-in technique by adding a small modulation voltage *V*_mod_ with a frequency of 4777 Hz to the bias voltage. All d*I*/d*V* spectroscopy curves were acquired by switching off the feedback loop at *V*_stab_ and *I*_stab_ and averaging several single d*I*/d*V* spectra. The in-gap maps have been obtained in multipass mode by first measuring each line in constant-current mode (at *V* and *I*) and then scanning each line again with the feedback off at a different tip–sample distance (by adding *z*_offset_) and a different value for the sample bias *V*_meas_.

The following measurement parameters were used for the data presented in the main figures: Figure 1a,b: *V* = −40 mV, *I* = 1 nA, *T* = 4.2 K, spin-polarized tip; f: *V* = −15 mV, *I* = 1 nA, *T* = 1.3 K. Figure 2: all *T* = 1.3 K, *V*_mod_ = 50 μV; b,c: spin-polarized tip, *V*_stab_ = +5 mV, *I*_stab_ = 5 nA; e,f: superconducting tip, *V*_stab_ = +4 mV, *I*_stab_ = 1 nA. Figure 4a: Multipass d*I*/d*V*: *T* = 1.3 K, *V* = −50 mV, *I* = 1 nA, *z*_offset_ = −150 pm, *V*_meas_ = +0.1 mV, *V*_mod_ = 50 μV. Figure 5d: Multipass d*I*/d*V*: *T* = 1.3 K, *V* = −50 mV, *I* = 1 nA, *z*_offset_ = −150 pm, *V*_meas_ = 0 mV, *V*_mod_ = 50 μV; inset constant-current SP-STM: *T* = 4.2 K, *I* = 1 nA, *V* = 4 mV.

### Theoretical Calculations

For a translation-invariant system, the Hamiltonian in eq 1 can be expressed in momentum space as

$$H_{k}=\sum_{k\in \mathrm{BZ}}\{\psi_{k}^{†}[\epsilon_{k}\sigma_{0}\tau_{z}+\alpha_{k}\cdot \sigma \tau_{z}+\Delta \sigma_{0}\tau_{x}]\psi_{k}+\psi_{k}^{†}[S_{Q}\cdot \sigma \tau_{0}]\psi_{k+Q}+\mathrm{h.c.}\}$$ 2

where

$$\epsilon_{k}=2t_{0}(\cos(k_{1})+\cos(k_{2}))+2t_{1}\cos(k_{1}-k_{2})+2t_{2}\cos(k_{1}+k_{2})-\mu$$ 3

$$\alpha_{k}=2\left(\alpha_{0}\sqrt{\frac{1}{3}}(\sin(k_{2})-\sin(k_{1}))-\alpha_{1}\sin(k_{1}-k_{2}),\;\alpha_{0}\sqrt{\frac{2}{3}}(\sin(k_{2})+\sin(k_{1}))+\alpha_{2}\sin(k_{1}+k_{2}),0\right)$$ 4

$$S_{Q}=\frac{1}{\sqrt{M_{1}M_{2}}}\sum_{r}S(r)e^{-iQ\cdot r}$$ 5

Here, *M*_1_ and *M*_2_ denote the size of the magnetic unit cell along the chosen lattice vectors ***a***_1_ and ***a***_2_, respectively. The parameters used in the main text are μ, *t*_0_, *t*_1_, *t*_2_, α_0_, α_1_, α_2_, *J* = (−1.05, 1.0, 1.0, 0.95, 0.077, 0.077, 0.077, 2.65)­Δ. These parameters are chosen to match the experimental observations, with the experimentally observed EMR signal constraining α, the observed localization length of the edge modes constraining the ratios of the hopping parameters and the superconducting order parameter, and the requirement of edge modes only emerging at [001]-edges constraining the ratios of the hopping parameters and the choice of chemical potential. No fine-tuning was necessary to obtain a TNPSC phase, which generically emerges over a large region of parameter space with *J* > Δ (see Figure S10). The sinusoidal and inhomogeneous spin spirals, with the spin **S**(**r**) at site **r** = (*x*, *y*) lying in the *xz*-plane, are described by

$$S(x,y)=N[(1-D)\cdot (\sin(Qx),0,\cos(Qx))+D\cdot (\sin(\theta_{\mathrm{inh}}),0,\cos(\theta_{\mathrm{inh}}))]$$ 6

where *Q* is the spiral wavevector, *D* ∈ [0, 1] represents the extent of the spiral’s inhomogeneity, and *N* is a normalization constant, such that |*S*(**r**)| = 1. Outside of the magnetic island, we rescaled the energies to ensure that the superconducting coherence peaks inside and outside the magnetic region lie at the same energy of ±Δ. The spatial dependence of the inhomogeneity is given by

$$\theta_{\mathrm{inh}}=\arcsin\left[\tanh\left(\frac{x-x_{l}}{w}\right)+\tanh\left(\frac{x-x_{r}}{w}\right)\right]$$

where *x*_*l*_ and *x*_*r*_ denote the closest in-plane regions left and right of *x*. Further, *w* is the amount of broadening, i.e., for *w* → 0, the domain wall becomes more and more steep.

### Topological Nodal Point Charges

The Hamiltonian in eq 1 has effective time-reversal T = τ_0_ σ_*y*_ λ_*z*_ *K*, charge C = τ_y_ σ_*y*_ λ_0_ *K*, and chiral S = τ_y_ σ_0_ λ_*z*_ *K* symmetries. Here, the τ_*a*_, σ_*b*_, and λ_*c*_ refer to the Pauli matrices in particle-hole, spin, and sublattice space, respectively. Their squares are given by $T^{2}=-I$ , $C^{2}=I$ , $S^{2}=I$ , resulting in the symmetry class DIII. By transforming the Hamiltonian to the eigenbasis of S , we obtain an off-diagonal matrix,

$$\begin{array}{rlll} U^{†}SU & =\left(\begin{array}{ll} 1 & 0\\ 0 & -1 \end{array}\right),\;H_{k}^{\prime } & =U^{†}H_{k}U & =\left(\begin{array}{ll} 0 & h_{k}\\ h_{k} & 0 \end{array}\right). \end{array}$$ 7

We can use the eigenenergies *E*_*n* **k**_ and eigenvectors |*n*_**k**_⟩ of *H*_**k**_^′^ to define

$$Q_{k}=\sum_{n}|n_{k}\rangle \mathrm{sgn}(E_{nk})\langle n_{k}|=\left(\begin{array}{ll} 0 & q_{k}\\ q_{k} & 0 \end{array}\right)$$ 8

The characteristic angle θ_**k**_ is obtained from *e* ^iθ_**k**_^ = det­(*q*_**k**_). The topological charge of a nodal point is then given by the winding number of the characteristic angle around the nodal point, i.e.,

$$\nu =\frac{1}{2\pi i}\oint dk\cdot \mathrm{Tr}[q_{k}^{-1}\nabla q_{k}]$$ 9

In Figure S6, we present the characteristic angle for the parameter set used in the main text. Here, nodal points with positive (+1) are shown in orange; those with negative charge (−1) are shown in green.

### Interplay of Conventional and Induced RSO Coupling

To understand the origin of the induced RSO coupling, we execute a local gauge transformation on the Hamiltonian discussed above

$$U_{r}=\exp\left(\frac{i}{2}\theta_{r}\;\sigma \cdot {\hat{n}}_{r}\right)$$ 10

with

$$\cos(\theta_{r})=\frac{S_{r}}{\left|S_{r}\right|}\cdot \hat{z},\;{\hat{n}}_{r}=\frac{S_{r}\times \hat{z}}{\left|S_{r}\times \hat{z}\right|}$$ 11

in spin space, such that *U*_**r**_^†^(**S**_**r**_·**σ**)*U*_**r**_ = *S*σ^z^. Here, the gauge transformation acts on the electronic creation and annihilation operators as *c*_**r**_ = *U*_**r**_ *f*_**r**_, where *c*_**r**_ = (*c*_**r**,*↑*_, *c*_**r**,*↓*_), which transforms the parts of the Hamiltonian describing the hopping and the magnetic structure as

$$\sum_{r,\delta }c_{r}^{†}[t_{\delta }\sigma_{0}-i\alpha_{\delta }(\delta \times \sigma {)}^{z}]c_{r+\delta }+J\sum_{r}c_{r}^{†}(S_{r}\cdot \sigma )c_{r}$$ 12

$$=\sum_{r,\delta }f_{r}^{†}U_{r}^{†}[t_{\delta }\sigma_{0}-i\alpha_{\delta }(\delta \times \sigma {)}^{z}]U_{r+\delta }\;f_{r+\delta }+JS\sum_{r}\;f_{r}^{†}\sigma^{z}f_{r}$$ 13

while leaving the other terms unchanged. This takes the spin spiral system with a homogeneous RSO coupling into an effective ferromagnetic system with a spatially varying RSO coupling, allowing us to study the interplay between the noncollinear spin texture and the conventional RSO coupling. Therein, the total Rashba spin–orbit coupling *RSO*_*t*_ is found as the off-diagonal entry of the matrix *U*_**r**_^†^[*t*_**δ**_σ_0_ – iα_**δ**_(**δ** × **σ**)^*z*^]*U*_**r**+**δ**_. We note that a right-rotating spiral with spiral vector **Q** and RSO coupling α possesses the same electronic structure as a left-rotating spiral with spiral vector −**Q** and RSO coupling −α.

The interplay between the conventional and induced RSO results in an interesting effect: (i) considering the termination angle dependence of the edge modes in Figure 5, we find that the maximum intensity is shifted from 0° and 180° by 8°, and (ii) there exists a second weaker intensity maximum at 243°. The edge mode at this second maximum decays more slowly. Hence, we show once again in Figure S8b the dependence of the edge mode intensity on the termination angle, but here we integrated over a few sites next to the edge, so that this second maximum can be seen more clearly. The existence of two intensity maxima arises from the fact that there are two sets of edge modes connecting the nodal points, one connecting the nodal points through *k*_*y*_ = 0 and the other through *k*_*y*_ = π/*a*. The intensity maximum at θ = 172° stems from the former, the one at θ = 243° from the latter, which can be seen in the dispersions shown in Figure S8d,e, respectively. At θ = 82°, the edge mode through *k*_*y*_ = 0 is most dispersive, which can be seen in the dispersion shown in Figure S8c. Due to the nonzero *RSO* coupling α in our model, these two edge modes evolve differently with a changing termination angle θ. Moreover, the shift of the larger intensity maximum away from θ = 0° and 180° is a direct consequence of the induced RSO coupling. This is shown in Figure S8, where we plot the zero-energy LDOS as a function of the termination angle θ and the spiral wavelength. We find that with increasing spiral wavelength, the intensity maxima move to 0° and 180°. Since the induced RSO coupling decreases with increasing spiral wavelength, we can conclude that a shift of the intensity maximum away from θ = 0° and 180° arises directly from the induced RSO coupling.

## Supplementary Material



## Data Availability

The data that support the findings of this study are available from the corresponding authors upon reasonable request.

## Notes

Contrast mechanism of the EMR effects; spin spiral profiles with different ratios between TMR and EMR; antiphase of the spin-resolved intensities of the positive and negative energy coherence peak; detailed view on the in-gap oscillations related to the EMR-effect; additional theoretical parameter sets; results for an inhomogeneous spin spiral; characteristic angle and further edge dispersions; line spectroscopy across a topological edge state; termination dependence of the spin spiral; the sample system at a finite magnetic field; phase diagram of the spin spiral system in the (μ, *J*)-plane; and zero-energy LDOS of the Fe/Ta island for topological and trivial parameter sets (PDF)
