# Supplementary material for: The Noncollinear Path to Two-Dimensional Topological Superconductivity
Source: ACS Nano. 2025 Oct 9;19(41):36215–22. doi: 10.1021/acsnano.5c07565 (PMC12548338; doi:10.1021/acsnano.5c07565)
Supplement: Supplementary file 1 [file nn5c07565_si_001.pdf]

Supplementary Information for: The Non-collinear Path to Two-dimensional Topological Superconductivity

Reiner Brüning,[1,*] Jasmin Bedow,[2] Roberto Lo Conte,[1,3]
Kirsten von Bergmann,[1,†] Dirk K. Morr,[2] and Roland Wiesendanger[1]
[1]*Department of Physics, University of Hamburg, 20355 Hamburg, Germany*
[2]*Department of Physics, University of Illinois Chicago, IL 60646, USA*
[3]*Zernike Institute for Advanced Materials, University of Groningen, 9747 AG Groningen, The Netherlands*

Note S1. Contrast mechanism of the EMR effects.
Figure S1. Spin spiral profiles with different ratios between TMR and EMR.
Figure S2. Anti-phase of the spin-resolved intensities of the positive and negative energy coherence peak.
Figure S3. Detailed view on the in-gap oscillations related to the EMR-effect.
Figure S4. Additional theoretical parameter sets.
Figure S5. Results for an inhomogeneous spin spiral.
Figure S6. Characteristic angle and further edge dispersions.
Figure S7. Line spectroscopy across a topological edge state.
Figure S8. Termination dependence of the spin spiral.
Figure S9. The sample system at finite magnetic field.
Figure S10. Phase diagram of the spin spiral system in the (μ, J)-plane.
Figure S11. Zero-energy LDOS of the Fe/Ta island for topological and trivial parameter sets.

Note S1.
Contrast mechanism of the EMR effects.

As explained in the main text the EMR contribution can originate from two effects. In the first case, TAMR, the local magnetization direction has an impact on the electronic states due to spin-orbit coupling [1, 2]. This manifests in STM measurements as a different signal of out-of-plane magnetized sample positions as compared to positions with local in-plane magnetization, resulting in a pattern with half of the magnetic period. The NCMR arises when the angle between magnetic moments spatially varies and was first identified in a system with localized non-collinear magnetic skyrmions in a collinear ferromagnetic background, where the spin mixing leads to different local electronic states for the two cases [3]. For a homogeneous spin spiral, the nearest neighbor angle is constant for the entire period, however, when the spin spiral is inhomogeneous the local angle between the spins varies with half the magnetic period, giving rise again to a signal modulation of that period in STM measurements [3].

* Email: rbruenin@physnet.uni-hamburg.de
† Email: kirsten.von.bergmann@physik.uni-hamburg.de

[1] K. von Bergmann, M. Menzel, D. Serrate, Y. Yoshida, S. Schröder, P. Ferriani, A. Kubetzka, R. Wiesendanger, and S. Heinze, Tunneling Anisotropic Magnetoresistance on the Atomic Scale, Physical Review B **86**, 134422 (2012).
[2] M. Hervé, B. Dupé, R. Lopes, M. Böttcher, M. D. Martins, T. Balashov, L. Gerhard, J. Sinova, and W. Wulfhekel, Stabilizing Spin Spirals and Isolated Skyrmions at Low Magnetic Field Exploiting Vanishing Magnetic Anisotropy, Nature Communications **9**, 1015 (2018).
[3] C. Hanneken, F. Otte, A. Kubetzka, B. Dupé, N. Romming, K. von Bergmann, R. Wiesendanger, and S. Heinze, Electrical Detection of Magnetic Skyrmions by Tunnelling Non-Collinear Magnetoresistance, Nature Nanotechnology **10**, 1039 (2015).

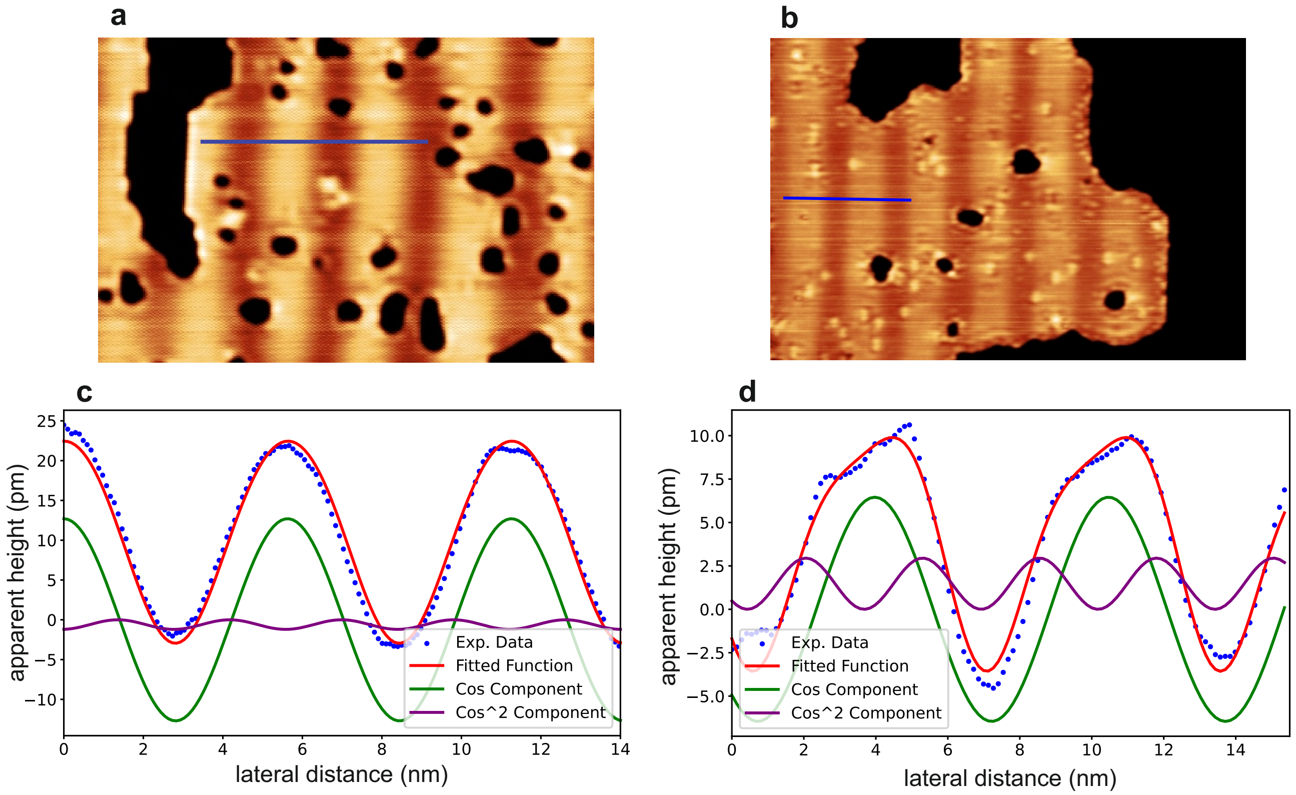

Figure S1. **Spin spiral profiles with different ratios between TMR and EMR.** **(a)** SP-STM constant-current image displaying a zoom-in into the same sample area as shown in Fig. 1a of the main text. **(b)** SP-STM constant-current image displaying an area of the same island as shown in Fig. 5d of the main text measured with a different tip and bias voltage than in **(a)**. **(c)**,**(d)** The blue dots represent data points corresponding to the line profile indicated by the blue line in **(a)** and **(b)**, respectively. The line profiles clearly show the spin spiral period due to the TMR. Both effects explained in Section 1 can in first approximation be modelled as $\cos^2$ function. Experimentally we can not distinguish which effect is dominating, however, in **(c)** we see a symmetric peak shape in the line profile, which leads to the conclusion of a negligible influence of the EMR effect in this measurement. To quantify the contributions of TMR and EMR in this experimental spin spiral profile, we performed a fit considering a cosine contribution for the TMR (green line) and a $\cos^2$ function for the EMR (purple line) related contributions to the signal. The red curve represents the fitted function and nicely reproduces the experimentally measured profile. The individual components manifest a dominating TMR contribution in this measurement. In **(d)**, we see a clear asymmetric peak shape in the line profile, which leads to the conclusion of a non-negligible influence of the EMR effect in this measurement. We performed the same fit to identify the contributions of TMR and EMR. The individual components of the fit offer a dominating TMR contribution but the ratio of the amplitudes between EMR and TMR significantly changed compared to **(c)**. Due to a canted tip magnetization direction, there is a phase shift between the TMR and EMR contributions. The resulting fitted function reproduces the experimentally measured profile and confirms a measurable EMR effect for the established spin spiral. Measurement parameters: Cr-tip, **(a)** $T = 4.2$ K, $I = 1$ nA, $V = -40$ mV; **(b)** $T = 1.3$ K, $I = 1$ nA, $V = 4$ mV.

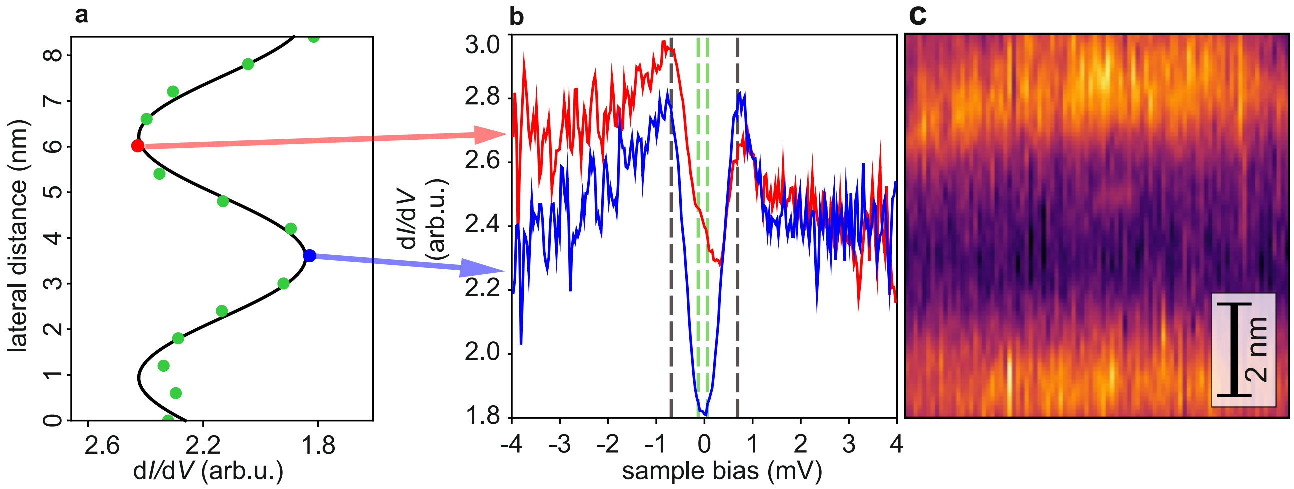

Figure S2. **Anti-phase of the spin-resolved intensities of the positive and negative energy coherence peak.** **(a)** Same panel as in Fig. 2c of the main text. **(b)** Two of the extreme data points (indicated in red and blue) have been selected from **(a)** and the corresponding spin-polarized $\mathrm{d}I/\mathrm{d}V$ spectra again demonstrate that the coherence peaks at positive and negative energy are both spin-polarized but with opposite sign. **(c)** A spin-resolved in-gap $\mathrm{d}I/\mathrm{d}V$ map obtained at $V = 0.1$ mV exhibiting the spin spiral period originating from the TMR effect. Measurement parameters: spin-polarized tip, $T = 1.3$ K. **a,b** See main text; **(c)** Multi-pass experiment: $V_{\mathrm{stab}} = +4$ mV, $I_{\mathrm{stab}} = 1$ nA, $z_{\mathrm{offset}} = 0$ pm, $I_{\mathrm{meas}} = 1$ nA, $V_{\mathrm{meas}} = +0.1$ mV, $V_{mod} = 50$ µV.

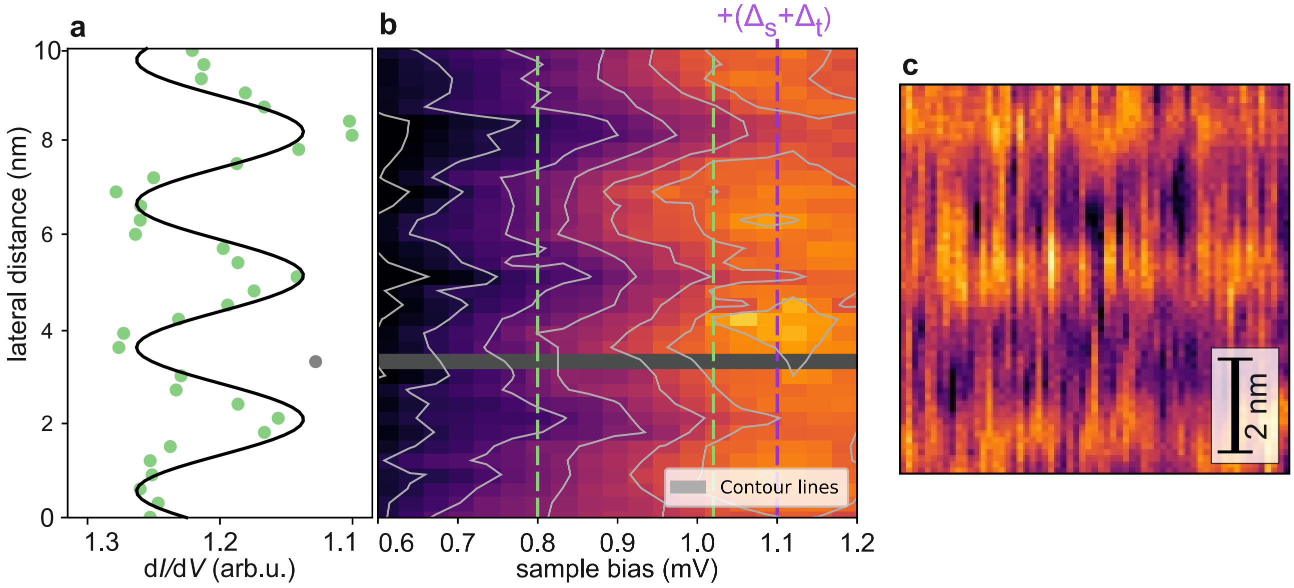

Figure S3. **Detailed view on the in-gap oscillations related to the EMR-effect.** **(a)** Same as in the main text, see Fig. 2F. **(b)** Enlarged view of the in-gap $\mathrm{d}I/\mathrm{d}V$ signal of Fig. 2e close to the coherence peak at positive bias voltage; the light grey lines represent contour lines and highlight the periodicity of the EMR. The horizontal dark grey line indicates one spectrum that was not considered for the contour line plot due to a momentary instability of the junction during data acquisition. **(c)** Spin-averaged in-gap current map at $V = 0.1$ mV exhibiting half the spin spiral period, i.e., originating from the EMR, obtained by using multi-pass experiments as described in the methods. Measurement parameters: superconducting tip, $T = 1.3$ K. **a,b** See main text; **(c)** $V_{\mathrm{stab}} = -50$ mV, $I_{\mathrm{stab}} = 1$ nA, $z_{\mathrm{offset}} = -150$ pm, $I_{\mathrm{meas}} = 1$ nA, $V_{\mathrm{meas}} = +0.1$ mV, $V_{mod} = 50$ µV.

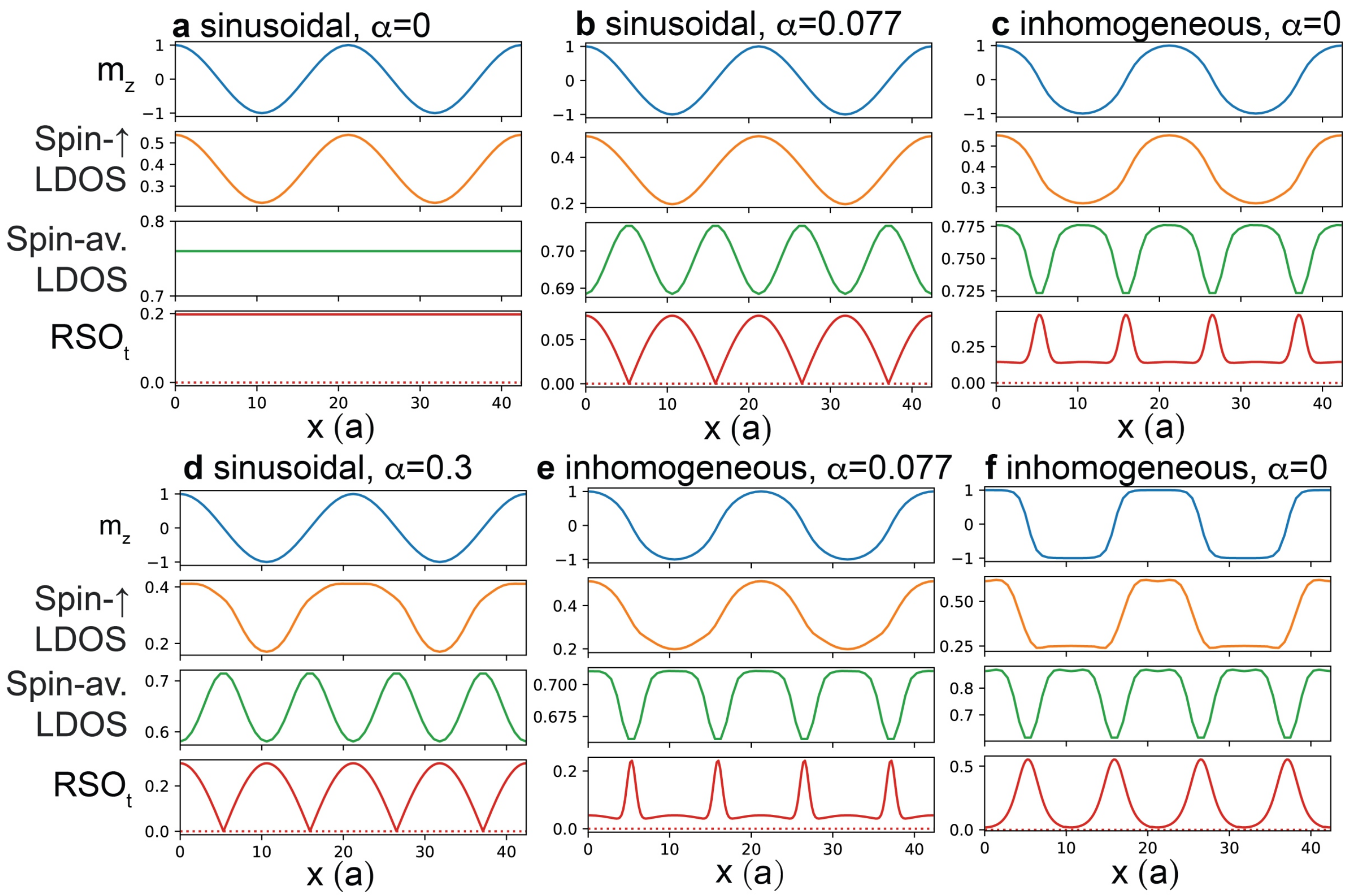

Figure S4. **Additional theoretical parameter sets.** We show the out-of-plane magnetization (blue), spin-↑ LDOS (orange), spin-averaged LDOS (green) and the absolute value of the total Rashba SOC along the relevant bonds over two spiral periods **(a)** for no spiral inhomogeneity $D = 0$ and no Rashba spin-orbit coupling $\alpha = 0.0\Delta$, **(b)** for no spiral inhomogeneity D and small Rashba spin-orbit coupling $\alpha = 0.077\Delta$ and **(c)** for small spiral inhomogeneity $D = 0.25$ and no Rashba spin-orbit coupling $\alpha = 0$, which all lead to sinusoidal oscillations in the spin-↑ LDOS. Further, we show the non-applicable cases **(d)** with no spiral inhomogeneity $D = 0$ and high Rashba spin-orbit coupling $\alpha = 0.3\Delta$, **(e)** for small spiral inhomogeneity $D = 0.25$ and small Rashba spin-orbit coupling $\alpha = 0.077\Delta$ and **(f)** high spiral inhomogeneity $D = 1$ and no Rashba spin-orbit coupling $\alpha = 0.0\Delta$. For the cases with non-zero D, we used different $\mu, J = (-1.0, 2.7)\Delta$. In all cases, the red dashed line in the panels showing RSO_t denotes where the total Rashba SOC is equal to zero. In **(a)**, **(c)**, **(f)**, we show the total Rashba SOC along horizontal $(1\bar{1}0)$-bonds, in **(b)**, **(d)** along vertical (001)-bonds, and in **(e)** along diagonal bonds.

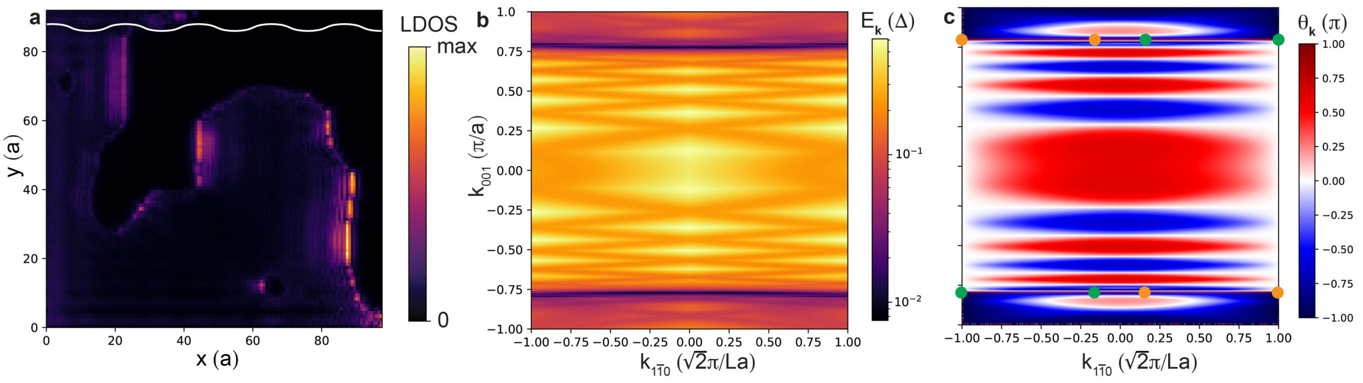

Figure S5. **Results for an inhomogeneous spin spiral.** **(a)** Theoretical spin-averaged zero-energy LDOS for a Fe island of the same size and shape as that shown in Fig. 5d. The white line represents the m_z-component of the spin spiral along the $[1\bar{1}0]$-direction. **(b)** Electronic dispersion and **(c)** characteristic angle $\theta_\mathbf{k}$ in the magnetic Brillouin zone with the nodal points marked according to positive (orange) and negative (green) charge. The parameters used are the same as in the main text except that here $\mu, J = (-1.0, 2.7)\Delta$.

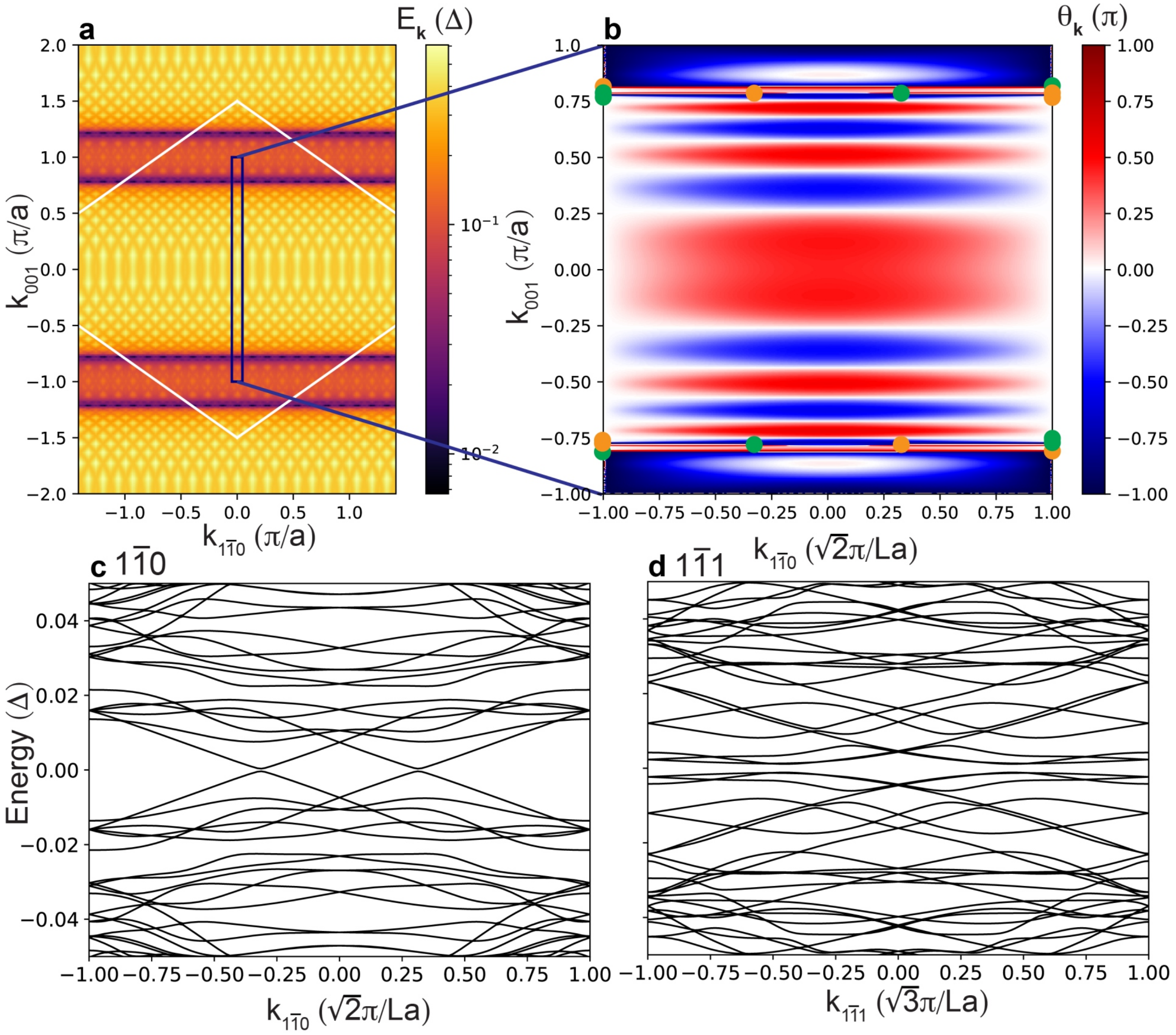

Figure S6. **Characteristic angle and further edge dispersions.** **(a)** Electronic dispersion in reciprocal space, where the hexagonal structural Brillouin zone (white) and the rectangular magnetic Brillouin zone (blue) are highlighted. **(b)** Characteristic angle $\theta_{\mathbf{k}}$ in the magnetic Brillouin zone with the nodal points marked according to positive (orange) and negative (green) charge. **(c)** Electronic dispersion on a ribbon along the $[1\bar{1}0]$ direction. **(d)** Electronic dispersion on a ribbon along the $[1\bar{1}1]$ direction.

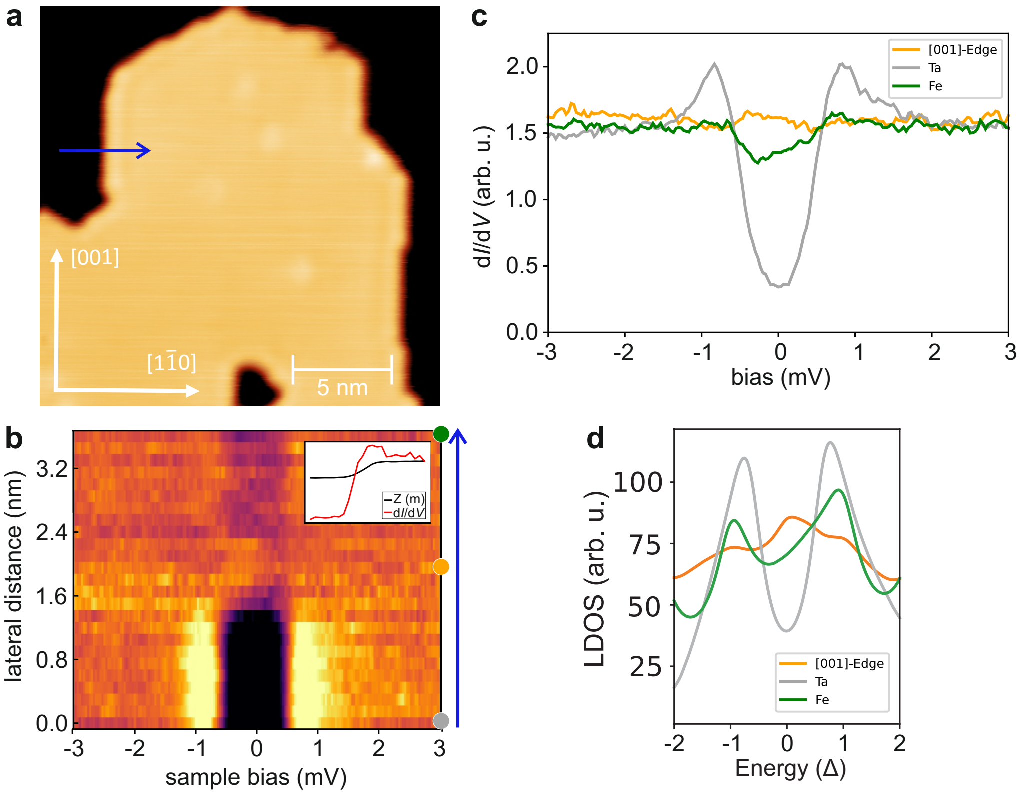

Figure S7. **Line spectroscopy across a topological edge state.** **(a)** A constant-current STM image of the identical island, as the one discussed in the main text (see Fig. 5d), is depicted. **(b)** Line spectroscopic measurement that was performed above a [001]-edge at the location marked by the blue arrow in **(a)**. Once again, a bright feature at the boundary between the Ta and Fe-bulk region is noticeable, highlighting the increased number of in-gap states due to the presence of an edge state. The inset within figure **(b)** displaying both the Z(m)- and dI/dV-channel at $V = 0$ V during the line spectroscopy. In **(c)**, three single-point spectra from the measurement in **(b)** on bare Ta, the [001]-edge and the Fe bulk area, located as indicated by the colorized circles in **(b)**, are presented. An enhanced signal around the Fermi level is observable in the spectrum at the [001]-edge, as expected for the topological edge state. Measurement parameters: superconducting tip, $T = 1.3$ K. **(a)** $I = 1$ nA, $V = 10$ mV; **(b)**,**(c)** $I_{stab} = 1$ nA, $V_{stab} = 4$ mV, $V_{mod} = 50$ µV. **(d)** Convoluted theoretical LDOS for the [001]-edge (orange), and the Ta (grey) and Fe (green) layers, taken from Fig. 4e and c, respectively. These data are convoluted with a Lorentzian of width 0.5Δ, in order to allow a comparison to the experimental data in panel c. The orange and green curve are shifted, such that the energy integral of all three curves for $E \in [-\Delta, \Delta]$ are the same.

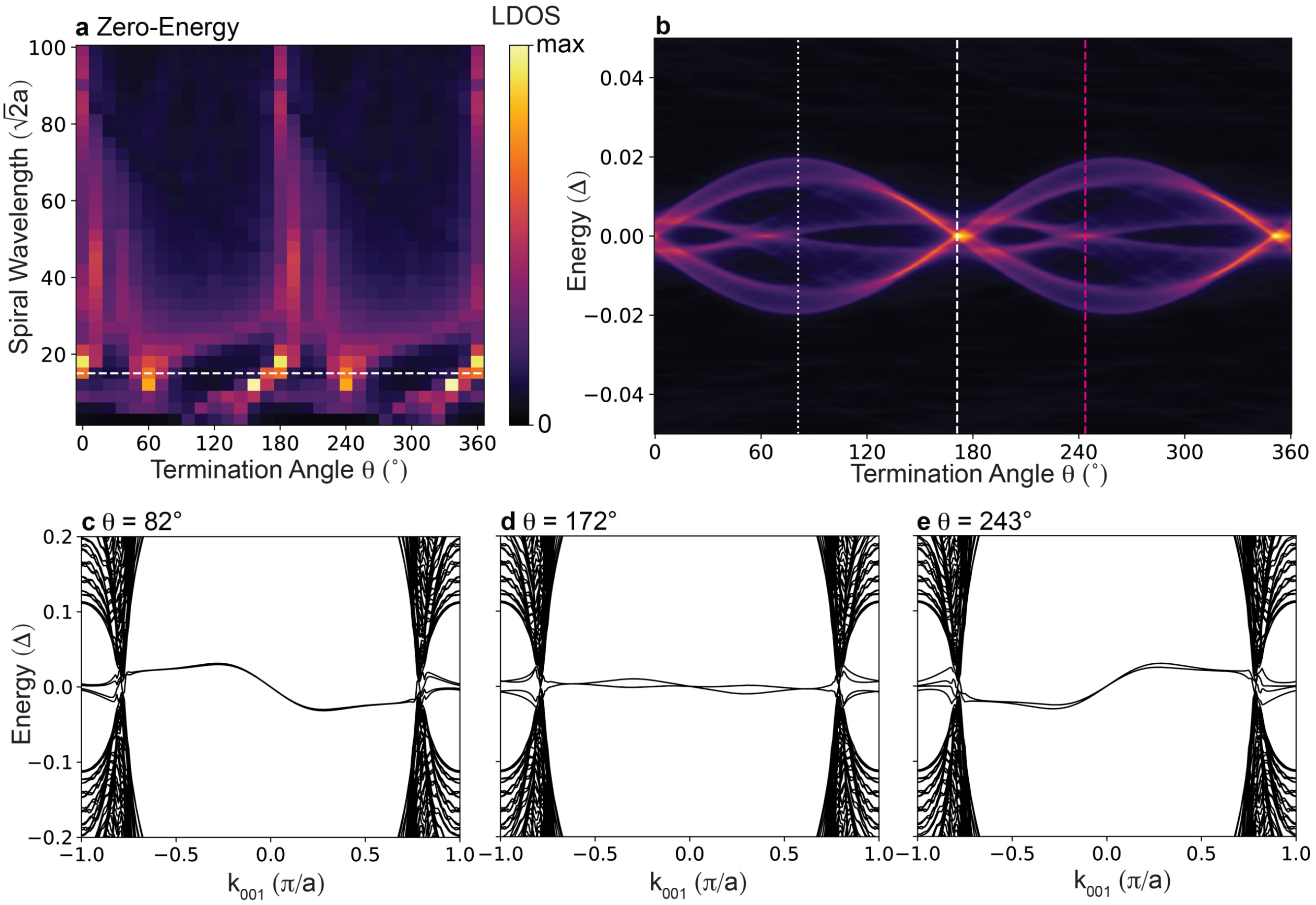

Figure S8. **Termination dependence of the spin spiral.** **(a)** Zero-energy LDOS as a function of the spiral termination angle θ and the spiral wavelength. For large spiral wavelengths, the maxima in the intensity move to 0° and 180°. **(b)** Low-energy LDOS as a function of the termination angle for the spiral wavelength in the text, averaged over 10 sites at the boundary of the topological region, to account for more slowly decaying modes. Band structure of the **(c)** most dispersive edge-mode for $\theta = 82^\circ$, **(d)** least dispersive edge mode for $\theta = 172^\circ$ and **(e)** second least dispersive for $\theta = 243^\circ$ at the left edge. The latter two create the maxima in panel b) through the very flat modes **(d)** between the nodal points and **(e)** outside the nodal points through the boundary of the Brillouin zone.

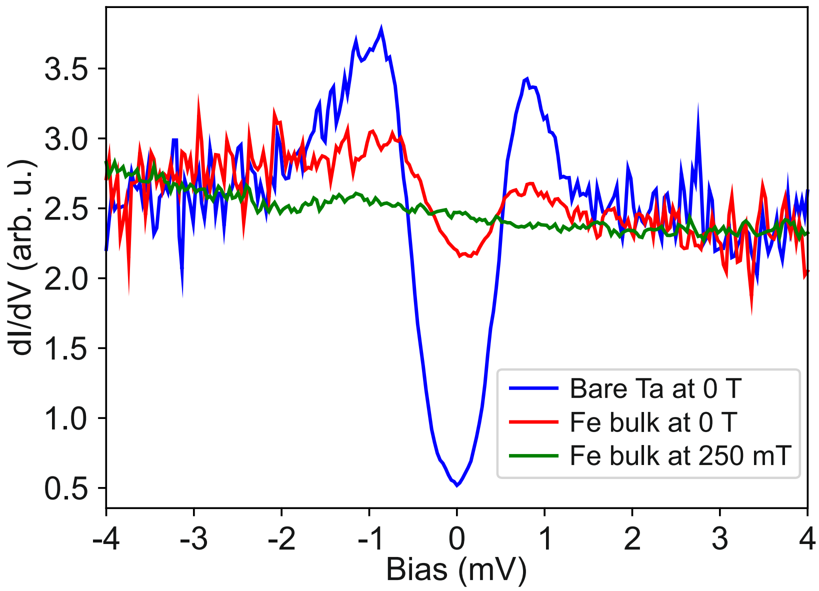

Figure S9. **The sample system at finite magnetic field.** Two single-point spectra are presented on bare Ta and the Fe bulk at 0 T and one single-point spectrum on the Fe bulk area at 250 mT. The spectrum obtained at finite magnetic field offers an almost flat conductance indicating that the system transitioned into the normal conducting state. Measurement parameters: Cr-tip, $T = 1.3$ K, $I_{stab} = 1$ nA, $V_{stab} = 4$ mV, $V_{mod} = 50$ µV.

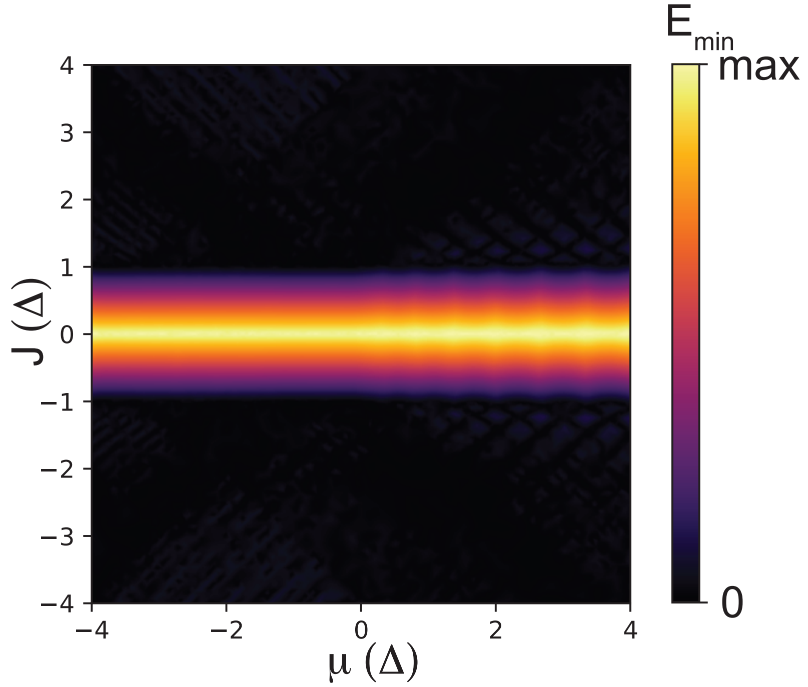

Figure S10. **Phase diagram of the spin spiral system in the** (μ, J)**-plane.** Minimum energy of the translation-invariant Fe/Ta system as a function of μ and J, with all other parameters the same as in the main text. The topological nodal-point phase emerges for $|J| > \Delta$.

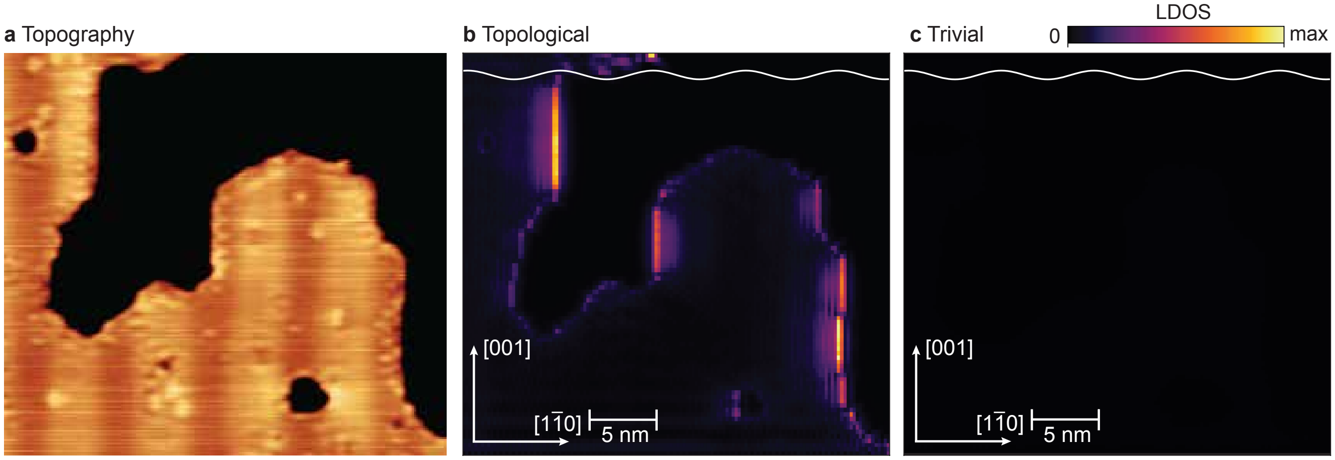

Figure S11. **Zero-energy LDOS of the Fe/Ta island for topological and trivial parameter sets.** **a** Topography of the Fe/Ta island, and zero-energy LDOS **b** from Fig. 5**e** of the main text, **c** for a trivial parameter set, where $J = 0.9\Delta$ and all other parameters the same as in the main text. As $J < \Delta$ for **c**, the system is in the trivial phase and gapped, possessing no in-gap state and thus not exhibiting any spectral weight along the edges of the island at low energies. The theoretical LDOS data were convoluted in energy with a Lorentzian of width $\Delta E = 0.01\Delta$.
